# A Genetic Screen for Functional Partners of Condensin in Fission Yeast

**DOI:** 10.1534/g3.113.009621

**Published:** 2013-12-20

**Authors:** Xavier Robellet, Lydia Fauque, Pénélope Legros, Esther Mollereau, Stéphane Janczarski, Hugues Parrinello, Jean-Pierre Desvignes, Morgane Thevenin, Pascal Bernard

**Affiliations:** * LBMC, CNRS UMR 5239, Ecole Normale Supérieure de Lyon, Université Claude Bernard Lyon 1, Lyon, France, F-69364; † MGX-Montpellier GenomiX, Institut de Genomique Fonctionnelle, Montpellier, France, F-34094

**Keywords:** condensin, fission yeast, synthetic lethality, mitotic chromosome condensation

## Abstract

Mitotic chromosome condensation is a prerequisite for the accurate segregation of chromosomes during cell division, and the conserved condensin complex a central player of this process. However, how condensin binds chromatin and shapes mitotic chromosomes remain poorly understood. Recent genome-wide binding studies showing that in most species condensin is enriched near highly expressed genes suggest a conserved link between condensin occupancy and high transcription rates. To gain insight into the mechanisms of condensin binding and mitotic chromosome condensation, we searched for factors that collaborate with condensin through a synthetic lethal genetic screen in the fission yeast *Schizosaccharomyces pombe*. We isolated novel mutations affecting condensin, as well as mutations in four genes not previously implicated in mitotic chromosome condensation in fission yeast. These mutations cause chromosome segregation defects similar to those provoked by defects in condensation. We also identified a suppressor of the *cut3-477* condensin mutation, which largely rescued chromosome segregation during anaphase. Remarkably, of the five genes identified in this study, four encode transcription co-factors. Our results therefore provide strong additional evidence for a functional connection between chromosome condensation and transcription.

Mitotic entry is characterized by the reorganization of long and entangled chromatin fibers into individualized, compact and rod-shaped mitotic chromosomes. This large-scale reorganization, called mitotic chromosome condensation, is essential for accurate chromosome segregation and is thought to serve at least two essential purposes: the removal of interchromosomal and interchromatids DNA catenations and the shortening of chromosome arms so that sister DNA molecules efficiently separate during anaphase. Although first described more than a century ago, the molecular mechanisms underlying mitotic chromosome condensation remain elusive.

From yeasts to human, mitotic chromosome condensation relies on two chromosomal components named topoisomerase II (topo II) and condensin (Baxter and Aragon 2012; Hirano 2012; Piazza *et al.* 2013). Condensin and/or topo II deficiency alters the structural integrity of chromosomes, leading to a failure to separate chromosomes arms and to the formation of chromatin bridges in anaphase. Although clear evidence now exists that condensin collaborates with topo II to remove catenations between chromatids (Baxter *et al.* 2011), how condensin associates with chromatin and, in this context, shapes mitotic chromosomes in a cell cycle−regulated manner remains poorly understood.

Condensin is a proteinaceous ring composed of two Smc ATPases, Cut3/Smc4/CAP-C and Cut14/Smc2/CAP-E, associated with three non-SMC auxiliary subunits (Hirano 2012; Piazza *et al.* 2013). Smc2 and Smc4 interact through their hinge domains, forming a V-shaped heterodimer with the two ATPase domains facing each other at the apices of two 50-nm-long coiled coil arms. The kleisin subunit Cnd2/Barren/CAP-H bridges the Smc2/Smc4 ATPase heads and recruits two additional subunits.

Condensin belongs to the family of structural maintenance of chromosomes (SMC) complexes, which includes the cohesin ring that mediates sister-chromatid cohesion. Like cohesin, condensin binds to chromosomes by encircling DNA, and this topological association appears important for chromosome condensation (Cuylen *et al.* 2011). On the basis of these observations, a structural model has been proposed whereby the role of condensin in chromosome condensation is to create and/or stabilize chromatin loops by encircling one or two chromatin fibers (Losada and Hirano 2005; Cuylen and Haering 2011). However, in human cells, ~80% of condensin I (the human counterpart of yeasts condensin) exchanges dynamically from mitotic chromosomes in early mitosis whereas the remaining ~20% are more stably bound (Gerlich *et al.* 2006), which suggests that at least two modes of interaction with chromatin may coexist and that condensin may drive chromosome condensation through a more dynamic and perhaps more complex mechanism. In line with this, condensin possesses an ATP-dependent DNA supercoiling activity stimulated by phosphorylation by the mitotic kinases cyclin-dependent kinase (CDK)1/Cdc2 and Polo (Kimura *et al.* 1998; St-Pierre *et al.* 2009; Piazza *et al.* 2013) and capable of driving decatenation by topo II during mitosis *in vivo* (Baxter *et al.* 2011). An enzymatic model proposes that condensin-mediated positive supercoiling of DNA is the driving force of mitotic chromosome condensation (Baxter and Aragon 2012). The different dynamics exhibited by chromosomal condensin complexes may reflect the coexistence of both an enzymatic and a more structural role.

With the notable exception of budding and fission yeasts, most eukaryotic species possess a second condensin complex, called condensin II, that differs from the canonical condensin (also known as condensin I) by a distinct set of three auxiliary, non-SMC proteins (Hirano 2012). Condensin I and II do not colocalize along the longitudinal axes of sister-chromatids, differ in the spatiotemporal regulation of their localizations, in their dynamics of chromatin association (condensin II being more stably bound), and in their respective contribution to mitotic chromosome formation (Hirano 2012). Thus, non-SMC subunits must play a key role in determining where, when, and how condensins associate with chromatin. However, the underlying mechanisms remain poorly understood.

Chromatin environment can also influence the association and/or activity of condensin complexes (Xing *et al.* 2008; Tanaka *et al.* 2012). Human condensin II binds monomethylated H4-K20 and tends to colocalize with this mark on chromosomes (Liu *et al.* 2010). Both human condensin I and fission yeast condensin bind histone H2A or its variant H2A.Z, and these interactions are stimulated by the phosphorylation of the kleisin Cnd2/Barren/CAP-H by Aurora-B kinase (Tada *et al.* 2011). Although condensin associates with nucleosomes, its distribution along chromosomes is not homogenous. Genome-wide binding studies in yeasts and chicken DT40 cells have shown that yeast condensin and vertebrate condensin I are enriched at centromeres and near highly expressed genes along chromosome arms (D’Ambrosio *et al.* 2008; Kim *et al.* 2013). Condensin binding near transfer RNA genes and at the 35S ribosomal DNA (rDNA) relies on the transcription factors TFIIIC and Acr1, respectively (D’Ambrosio *et al.* 2008; Nakazawa *et al.* 2008; Tada *et al.* 2011). Furthermore, a physical interaction has been described between condensin and TFIIIC in budding and fission yeasts (Haeusler *et al.* 2008; Iwasaki *et al.* 2010). Yet, transcription has been shown to preclude condensin binding (Clemente-Blanco *et al.* 2009, 2011), and condensin accumulates on chromatin during mitosis, when transcription is generally down-regulated. The biological significance of this apparent paradox is not yet known.

Potentially adding further complexity to the picture are reports that depletion of Smc4 or Smc2 delays, but does not prevent, the formation of compacted metaphase chromosomes in *Caenorhabditis elegans*, chicken, and human cells (Hudson *et al.* 2009). However, those condensin-depleted chromosomes lack structural integrity and form extensive chromatin bridges during anaphase. These observations led to the proposal that condensin’s primary function is to preserve the structural integrity of the mitotic chromosome during anaphase while an as-yet-identified activity called regulator of chromosome architecture (RCA) ensures the initial compaction of chromatin when CDK activity is high (Vagnarelli *et al.* 2006). However, the finding that 5% of the wild-type level of Smc2 persist in Smc2-depleted chicken DT40 cells (Ohta *et al.* 2010) raises the alternative possibility that traces of condensin suffice to drive condensation from prophase to metaphase but not for providing the stiffness required for chromosome segregation upon anaphase onset.

Regardless of the exact mechanisms, condensin most likely functions with multiple cofactors to associate with chromatin and to ensure its reorganization in a cell cycle−regulated manner. To gain insights into the mechanisms of condensin binding and mitotic chromosome condensation, we searched for factors that collaborate with condensin through an unbiased genetic screen in fission yeast. Using a tester strain bearing a thermosensitive allele of the Cut3/Smc4 ATPase (Saka *et al.* 1994), we isolated mutations resulting in synthetic lethality at the permissive temperature. As expected, our screen resulted in the identification of mutations in other condensin subunits. In addition, we identified a class of mutations affecting chromatin modifiers linked to transcription, consistent with a close functional interplay between condensin and the transcription machinery.

## Materials and Methods

### Media, molecular genetics, and strains

Media and molecular genetics methods were as described previously (Moreno *et al.* 1991). Complete medium was YES+A. The synthetic medium was PMG unless otherwise stated. Gene deletions were performed using a polymerase chain reaction (PCR)-based method, as described (Bahler *et al.* 1998). All deletions were confirmed by PCR. Strains used in this study are listed in Supporting Information, Table S1.

### Construction of the tester strain *nmt41-cut3 cut3-477*

The *cut3* open reading frame was PCR-amplified from genomic DNA using Pfu ultra DNA polymerase with primers GTTGTCGCGAACACACCTCTTTTCACGAC and TATACCGCGGCTTTGCGCAGATTTTACAG, digested with *Nru*I and *Sac*II (sites are underlined in primer sequences), and cloned into the replicative expression vector pJR2-41XL (Moreno *et al.* 2000). The recombinant plasmid, pREP41-cut3, was linearized by cutting within *ars1* with *Mlu*I and inserted into the genome of a thermosensitive *cut3-477* mutant strain, presumably at the *ars1* locus. A stable transformant that exhibited thiamine-dependent thermosensitivity for growth at 36° was selected and backcrossed to give rise to the tester strain *nmt41-cut3 cut3-477*. The genes *cut3-477* and *nmt41-cut3* segregated independently of each other in crosses.

### Mutagenesis and screening

The tester strain was mutagenized with ethylmethane sulfonate 2% (w/v) during one generation at 32° in EMM2 synthetic medium without thiamine, as described (Moreno *et al.* 1991). Mutagenized cells were plated on PMG medium without thiamine at 32°, and colonies were replicated onto PMG plus phloxine B without or with (20 μM) thiamine. Colonies that stained dark red onto PMG phloxine B plus thiamine at 32° were selected.

### Reverse Transcriptase (RT)-quantitative (q)PCR

Total RNA was extracted from 10^8^ cells by standard hot-phenol method. Reverse transcription was performed on 500 ng of total RNA using Superscript II (Invitrogen) and random hexamers. RT-qPCR analyses were performed with a Rotor-Gene PCR cycler.

### Genetic mapping

Genetic mapping was performed as described (Anders *et al.* 2008). Mutations were assigned to a given chromosome through homozygous *rec12Δ* crosses. Subchromosomal mapping, when necessary, was performed using *swi5Δ*.

### Immunofluorescence

Immunofluorescence was performed as described previously (Bernard *et al.* 2010) with the use of anti-α tubulin Tat1 antibody (Woods *et al.* 1989). Spindle length was measured with Image J software.

### Chromatin immunoprecipitation (ChIP)

Cells arrested in prometaphase at 17° were fixed with 1% formaldehyde for 30 min and processed for chromatin immunoprecipitation as described (Bernard *et al.* 2010) using the anti-GFP antibody A11122. Immune complexes were collected with Dynabeads protein A. Real time-qPCR analyses were performed using Rotor-Gene PCR cycler. Primers sequences are available upon request.

### Sequencing

Next-generation sequencing was performed by using Illumina HiSeq 2000. A total of 20 μg of genomic DNA was prepared from the mutant strains *slc129*, *slc174*, *slc185*, or *sup122*, sonicated using BioRuptor for 35 min at 320W (30 sec ON, 30 sec OFF) and processed using the TruSeq DNA Sample Prep Kit (Illumina). DNA samples were sequenced with a coverage varying between 180 and 230X and aligned to the *S. pombe* reference genome using the CASAVA 1.8 software. Single-nucleotide polymorphisms (SNPs) and single-nucleotide insertions or deletions were sought using CASAVA 1.8. Mutations of interest were confirmed by PCR amplification of candidate gene using Phusion DNA polymerase and Sanger sequencing of the PCR product.

## Results

### The condensin mutant *cut3-477* is defective for chromosome condensation at 32°

**Figure 1:**
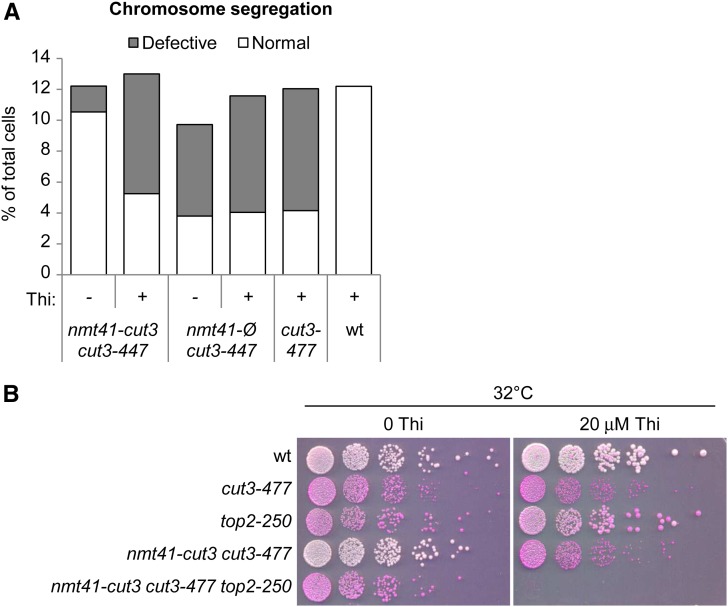
The *nmt41-cut3 cut3-477* tester strain. (A) Cell cultures in synthetic medium without thiamine (Thi) at 25° were split in two, and Thi (20 μM final) was added to one half. Cells were incubated at 25° for three doublings, shifted to 36° for three additional doublings, fixed, and stained with Hoechst 33342 to visualize DNA. Binucleated cells were examined for chromosome segregation defects (n > 100). (B) Cells grown in synthetic medium without Thi were serially diluted and spotted on synthetic medium plus phloxine B supplemented or not with Thi (20 μM final).

To identify factors that collaborate with condensin, we performed a genetic screen for mutants unable to survive when condensin is partly deficient. We chose the thermosensitive *cut3-477* mutation to weaken condensin because this mutation in the Cut3/Smc4 ATPase has been shown to cause synthetic lethality when combined with *top2-250* at the permissive temperature of 30° (Saka *et al.* 1994), suggesting that *cut3-477* mutant cells survive with a partly defective condensin complex. In agreement, we observed chromatin bridges and chromatin masses trailing on the mitotic spindle in *cut3-477* mutant cells cultured at 25°, 32° and 37° (Figure S1A). Growth of the *cut3-477* mutant strain at 32° (see Figure 1B) despite the presence of chromosome segregation defects in ~60% of anaphases may be explained by the resolution of those chromosome segregation defects by telophase, an event that might escape our detection focused on anaphase cells. We also assessed condensin binding to chromatin by ChIP. Cells expressing the GFP-tagged Cnd2 subunit of condensin were arrested in prometaphase by using the cryosensitive *nda3-KM311* mutation and processed for ChIP against GFP. Mitotic arrest was achieved with similar efficiency in *cnd2-GFP* and *cnd2-GFP cut3-477* strains, as indicated by the septation indexes and the percentages of cells showing Cnd2-GFP enriched in the nucleus (Figure S1B) (Sutani *et al.* 1999). In good agreement with recent results (Tada *et al.* 2011), we found that the binding of Cnd2-GFP to centromere, rDNA, and several sites distributed along chromosome arms was reduced in a *cut3-477* background (Figure S1B). These data indicate that condensin binding and mitotic chromosome condensation are impaired in a *cut3-477* genetic background at 25° and 32°, although the *cut3-477* mutant strain is alive at these temperatures. This most likely renders the *cut3-477* strain hypersensitive to additional nonlethal mutations that alter the condensation process, such as *top2-250*. Therefore, screening for mutations synthetically lethal with *cut3-477* may lead to the identification of factors required for mitotic chromosome condensation.

### Construction of a tester strain, *nmt41-cut3 cut3-477*

To screen for mutations synthetically lethal with *cut3-477*, we created the tester strain *nmt41-cut3 cut3-477*, which possesses the *cut3-477* mutation at the *cut3* locus plus a wild-type *cut3* gene whose expression is driven by the thiamine-repressible promoter *nmt41* (*n*o *m*essage in *t*hiamine) (Basi *et al.* 1993), inserted into the genome at an ectopic location. Because the *cut3-477* mutation is recessive (Saka *et al.* 1994), the tester strain should behave as a wild-type strain when grown in the absence of thiamine (*nmt41-cut3* expressed), but like a *cut3-477* mutant in the presence of thiamine (*nmt41-cut3* repressed). An asynchronous population of exponentially growing fission yeast cells contains ~10% of mitotic cells. As shown in Figure 1A, *nmt41-cut3 cut3-477* cells cultured in the absence of thiamine at 36° exhibited less than 2% of defective anaphases, but the frequency increased to 7% in the presence of thiamine, reaching a level similar to the *cut3-477* mutant. In contrast, the addition of thiamine to the growing medium did not modify the frequency of defective anaphases exhibited by *nmt41-Ø cut3-477* cells, bearing an empty *nmt41* plasmid. These data indicate that the *nmt41-cut3* gene complements, at least partly, the *cut3-477* mutation at 36° in the absence of thiamine, but not in the presence of 20 μM thiamine. Furthermore, using the tester strain we observed a thiamine-dependent synthetic lethal genetic interaction between *top2-250* and *cut3-477* at 32° (Figure 1B). Thus, the strain *nmt41-cut3 cut3-477* behaves as a wild-type strain when it is cultured in the absence of thiamine, but like a *cut3-477* condensin mutant strain in the presence of thiamine.

### Screening for mutations synthetically lethal with *cut3-477*

**Figure 2:**
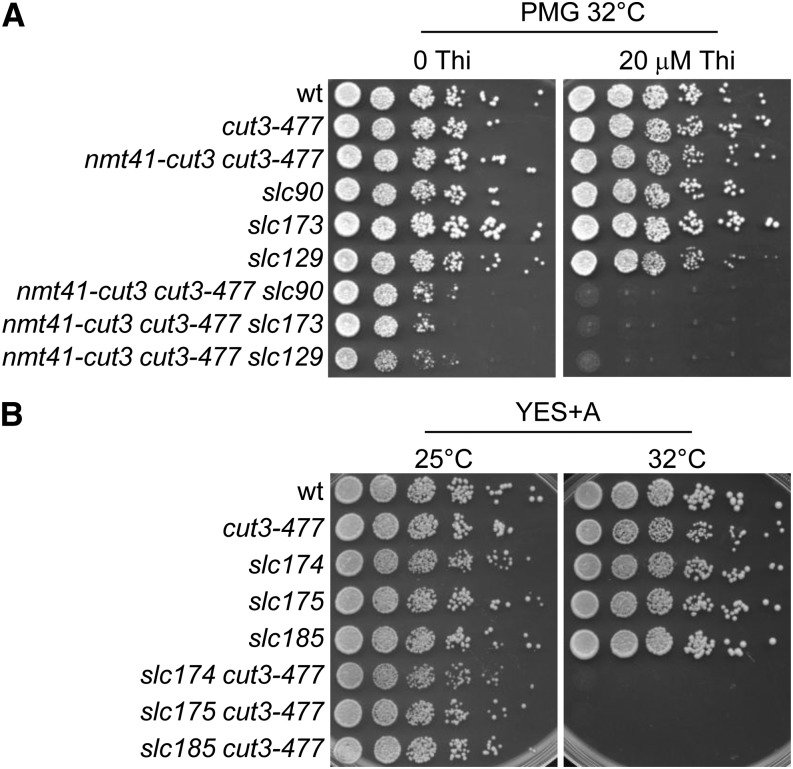
*slc* mutations are synthetically lethal with *cut3-477*. Strains of indicated genotypes were serially diluted and spotted onto (A) synthetic medium without thiamine (0 Thi) or supplemented with thiamine (20 μM final) to repress transcription of the *nmt41-cut3* gene, or (B) onto complete medium.

The tester strain was mutagenized with ethylmethane sulfonate and mutant colonies viable on synthetic medium without thiamine but dead on synthetic medium plus thiamine at 32° were selected. Mutants were subsequently backcrossed at least three times. The first backcross was performed with a wild-type strain (lacking both *nmt41-cut3* and *cut3-477*). This allowed us to eliminate secondary mutation in the *cut3-477* gene that were lethal at 32° because mutants bearing this type of mutation cannot produce *cut3-477* progenies viable at 32° in the absence of *nmt41-cut3*. This first backcross also allowed the elimination of mutations that conferred thiamine hypersensitivity, *i.e.*, mutants that produced progenies hypersensitive to thiamine regardless of *nmt41-cut3*. From ~180,000 colonies, 15 *slc* mutants (*s*ynthetically *l*ethal with *c*ut3) were selected (see Table 1). Representative examples are shown in Figure 2A. During the course of the backcrosses, we noticed that all *slc* mutants, except *slc129*, produced *cut3-477 slc* progenies devoid of the *nmt41-cut3* gene at 25°. Those *cut3-477 slc* double mutants were viable at 25° but dead at 32° (Figure 2B). Thus, the synthetically lethal interactions between the vast majority of *slc* mutations and *cut3-477* are thermo-dependent.

**Table 1:** Genes identified in the screen

Gene Name	*cut14*	*cnd1*	*cnd3*	*ark1*	*arp9*	*snf21*	*cph2*	*ulp2*	*nut2*
Function	Condensin Smc2	Condensin non-Smc	Condensin non-Smc	Aurora-B kinase	Swi/Snf and RSC chromatin remodeling	RSC chromatin remodeling	Clr6S Histone deacetylase	Sumo decon-jugating enzyme	Mediator
*slc* mutations	*slc71*	*slc175*	*slc184*	*slc190*	*slc127*	*slc129*	*slc174*	*slc185*	*sup122*
	*slc85*	*slc181*							
	*slc90*	*slc182*							
	*slc173*								
	*slc179*								
	*slc180*								
Allele names	*cut14-71*	*cnd1-175*	*cnd3-184*	*ark1-190*	*arp9-127*	*snf21-129*	*cph2-174*	*ulp2-185*	*nut2-122*
	*cut14-85*	*cnd1-181*							
	*cut14-90*	*cnd1-182*							
	*cut14-173*								
	*cut14-179*								
	*cut14-180*								
Protein	71: G397D	175: G667E	E195K	R241H	Q13*^a^*	E524K	C453Y	R81C	M1I
	85: D656N	181: G912D							
	90: T652M	182: R1117C							
	173: T652M								
	179: T652M								
	180: C631Y								

RSC, remodels the structure of chromatin.

a STOP codon.

### Genetic analyses

All *slc* mutations segregate 2:2 in crosses. They are recessive and organized into eight complementation groups, defining eight loci (Table 1). To test for allelism with expected hits, each *slc* locus was assessed for genetic linkage with chromosome condensation genes: condensin subunits *cut14*, *cnd1*, *cnd2*, and *cnd3*, *top2*, aurora-B and polo kinases, survivin, incenp, *pcs1*, and *mde4* (Tada *et al.* 2011). We observed a tight linkage between 3 *slc* loci (representing 10 mutations) and *cut14*, *cnd1*, or *cnd3*, and between a fourth *slc* locus and *ark1*, which encodes Aurora-B kinase. Sanger sequencing of those genes in candidate *slc* mutant backgrounds confirmed allelism. The 11 *slc* mutations were therefore renamed following the rule *gene name-mutation number*, *e.g.*, the *slc71* mutation in the *cut14* gene was renamed *cut14-71* (Table 1). For the remaining *slc127*, *slc129*, *slc174*, and *slc185* loci, we observed no genetic linkage with any tested condensation gene, raising the possibility that these four loci could correspond to unknown chromosome condensation genes.

### Phenotypic analyses

**Figure 3:**
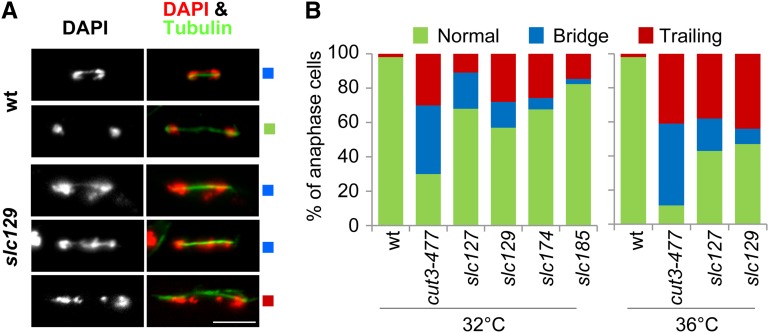
Defective chromosome migration during anaphase in *slc* mutant cells. (A) Chromosome segregation in cells fixed and processed for immunofluorescence against α-tubulin (Tubulin). DNA was stained with 4′,6-diamidino-2-phenylindole (DAPI). Bar: 5 μm. (B) Frequencies of chromosome segregation defects in late anaphase. Cells were fixed, processed for tubulin staining, and examined for chromosome segregation defects in late anaphase (spindle >5 μm, n > 100). Left panel: cells were cultured and fixed at 32°. Right panel: cells cultured at 32° were shifted at 36° for 2.5 hr and fixed at 36°.

*slc* mutations might alter mitotic chromosome condensation. To test this, we examined chromosome segregation in *slc* single-mutant cells. Mitotic cells were identified by the presence of a mitotic spindle and examined for chromosome segregation defects. In wild-type cells, the migration of centromeres to the opposite poles of the mitotic spindle upon anaphase onset, and the movement of chromosome arms that ensues, lead to the formation of a mini chromatin bridge, which rapidly disappears as cells progress into late anaphase (Tada *et al.* 2011). We observed mini chromatin bridges on short mitotic spindles with length comprised between 3 and 4 microns in wild-type cells (Figure 3A). However, these chromatin bridges disappeared as mitotic spindles increased to 5 microns in length, and passed this stage, chromosomes were almost always fully separated and clustered at the spindle poles (Figure 3, A and B). In sharp contrast, the four mutants, *slc127*, *slc129*, *slc174*, and *slc185*, exhibited frequent chromatin bridges and/or chromatin masses trailing on long anaphase spindle (>5 microns in length; Figure 3, A and B). Thus, chromosome migration during anaphase is altered in *slc127*, *slc129*, *slc174*, and *slc185* single-mutant cells.

Cut3 is an essential gene, and *slc* mutations may interfere with its expression. Similarly, a reduced expression of *top2* would most likely kill a *cut3-477* mutant strain. We therefore assessed by RT-qPCR the expression levels of each subunit of condensin and of *top2* in *slc127*, *slc129*, *slc174*, and *slc185* single-mutant cells cultured at 32° (Figure S2). We observed no obvious reduction in the steady state levels of either condensin or *top2* mRNA in any *slc* mutant, arguing against an indirect negative effect of *slc* mutations on the expression of these genes.

We assessed *slc* mutants for additional phenotypes. We observed no cryosensitivity for growth at 20° (data not shown). However, *slc127*, *slc129*, and *slc174*, as well as most *slc* mutants of the Cut14 subunit of condensin, exhibited thermosensitivity for growth at 36° (Figure S3A). Condensin takes part in the attachment of kinetochores to microtubules during mitosis, and in the signaling and/or repair of DNA damage during interphase, as indicated by the hypersensitivity to hydroxyurea (HU) and ultraviolet light of *cut14-Y1* and *cnd2-1* mutants (Aono *et al.* 2002; Akai *et al.* 2011; Tada *et al.* 2011). To test for a possible role of *slc* genes in these processes, we examined the growth of *slc* mutants in the presence of increasing amounts of thiabendazole, an inhibitor of microtubule polymerization, or in the presence of HU. The *cut14-71*, *slc127*, and *slc129* mutants, and to a lesser extent *ark1-190*, showed hypersensitivity to TBZ (Figure S3B). In addition, *slc127*, *slc129*, *slc174*, and *cnd1-175* were hypersensitive to HU (Figure S3C). However, we observed no hypersensitivity to ultraviolet radiation ranging from 50 to 100 J/m2 (not shown).

### Identification of *slc* mutations

The *arp9* gene was cloned by functional complementation of the *slc127* thermosensitive phenotype using the pUR19-B1 genomic library (Barbet *et al.* 1992). Sequencing *arp9* in the *slc127* mutant strain revealed a C37T mutation (numbering starting from the ATG) that created a premature stop codon. The *slc127* mutation was therefore renamed *arp9-127*. We failed to clone the three other *slc* genes by functional complementation. Those genes were identified by an alternative approach combining next-generation, whole-genome sequencing of *slc* mutants strain and genetic mapping of *slc* point mutations. We identified 109 ± 8 SNPs in each *slc* mutant strains (File S1, File S2, File S3). First, pairwise comparisons of the lists of SNPs using a home-made script allowed us to identify 5 ± 3 SNPs that were unique to each mutant background. Second, each *slc* mutation was assigned to one chromosome by cosegregation analyses (Anders *et al.* 2008), further refining the number of candidate SNPs to 3 ± 2 per mutant genome. Finally, candidate single-base mutations were identified by genetic linkage analyses and confirmed by Sanger sequencing (Table 1). *slc129* corresponds to *snf21*, *slc174* to *cph2*, and *slc185* to *ulp2*. Except for *snf21*, which is an essential gene, we confirmed that deleting *arp9*, *cph2*, or *ulp2* was synthetically lethal with *cut3-477* at 32° (Figure S4).

### *nut2* is a suppressor of *cut3-477*

**Figure 4:**
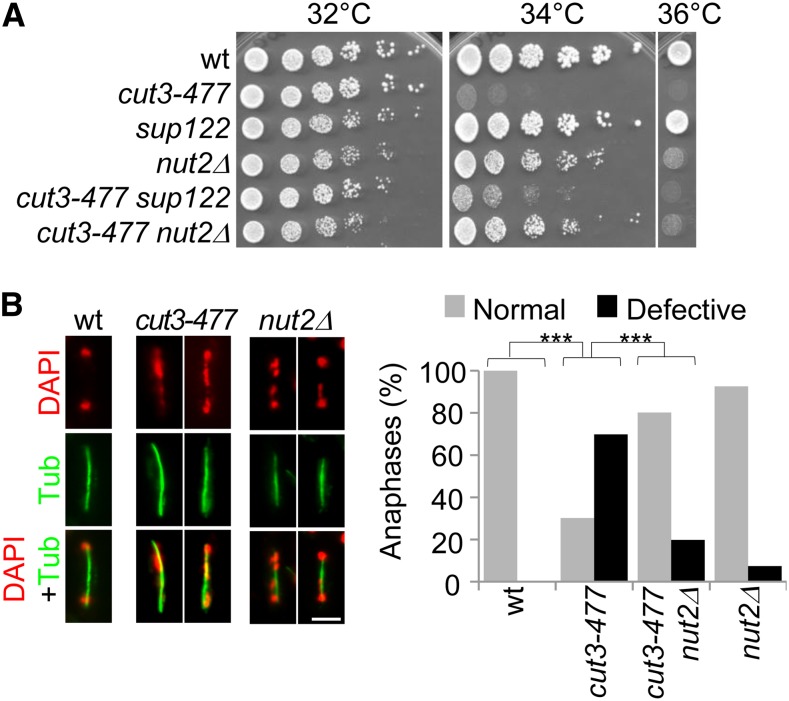
The lack of *nut2* partly compensates for *cut3-477* deficiency. (A) Cells of indicated genotypes were serially diluted and spotted onto complete medium. (B) Cells exponentially growing at 30° were shifted at 34° for one generation, fixed, and processed for immunofluorescence against α-tubulin (Tub) and DNA staining with DAPI. Bar, 5 μm. Chromosome segregation was assessed in late anaphase cells (spindle >5 μm, n > 100). ***P < 0.001, χ^2^ test.

The *nmt41-cut3 cut3-477* tester strain forms pink colonies at 32° on synthetic medium supplemented with phloxine B and 20 μM thiamine (Figure 1B). During the screening procedure, we picked a mutant colony that remained white on synthetic medium plus phloxine B plus thiamine at 32°, suggesting a possible suppressive effect on *cut3-477*. Backcrosses confirmed the presence of a suppressor mutation, named *sup122*, distinct from the *cut3* locus and independent of *nmt41-cut3*, which restored the growth of *cut3-477* at restrictive temperature (Figure 4A). The *sup122* mutation segregates 2:2 in crosses and is recessive. Genome resequencing of the *sup122* mutant strain combined with genetic mapping revealed a point mutation in the nonessential *nut2* gene (Table 1 and File S4), and we confirmed that deleting *nut2* restored growth of *cut3-477* at 34° (Figure 4A). To investigate the mechanism through which the lack of *nut2* partly suppressed the thermosensitivity of *cut3-477*, we assessed chromosome segregation in *cut3-477 nut2Δ* double-mutant cells at 34° (Figure 4B). The frequency of defective anaphases was markedly reduced in *cut3-477 nut2Δ* cells compared with *cut3-477*, although chromosome segregation was altered in ~7% of anaphases in *nut2Δ*. Thus, *nut2* is required for normal chromosome segregation and its deficiency rescues chromosome segregation when condensin is impaired by the *cut3-477* mutation.

## Discussion

To identify genes that potentially interact with condensin to mediate chromatin interaction and compaction, we performed an unbiased genetic screen for genetic interaction partners of the Cut3/Smc4 ATPase subunit of condensin in fission yeast. This screen identified novel mutations in the condensin subunits Cut14, Cnd1, and Cnd3, and in Aurora-B kinase, which regulates condensin binding to chromatin (Tada *et al.* 2011), demonstrating its efficiency in identifying *bona fide* condensation factors. In addition, we isolated mutations in *arp9*, *snf21*, *cph2*, and *ulp2*, four genes not previously implicated in mitotic chromosome condensation in fission yeast. We show that all these mutations cause chromatin bridges and/or segregation anomalies in anaphase, consistent with defective chromosome condensation. In the course of this screen, we independently identified *nut2Δ* as a suppressor of the thermosensitive growth and segregation defects caused by the condensin mutation *cut3-477*. Thus, Arp9, Snf21, Cph2, Ulp2, and Nut2 may play a role in mitotic chromosome condensation.

The presence of single alleles of *arp9*, *snf21*, *cph2*, or *ulp2* indicates that the screen is not saturated and therefore that additional genes synthetically lethal with *cut3* remain to be identified. Consistent with this, using high-throughput genetic interaction mapping Ryan *et al.* (2012) reported negative genetic interactions between *cut3* and at least 32 non-essential genes, including *ulp2*. Surprisingly, however, the screen failed to detect *arp9* and *cph2*, although *cph2* was present in the deletions library they used for screening.

The *cut14-71* mutation identified here confers hypersensitivity to TBZ. To the best of our knowledge, this is the first described mutation in condensin that confers pronounced TBZ hypersensitivity. The fact that among eight condensin mutants (seven *slc* alleles plus *cut3-477*), solely *cut14-71* exhibits this phenotype, suggests that this type of mutation may be rare. Pcs1 recruits condensin at the kinetochore (Tada *et al.* 2011), and like *cut14-71*, *pcs1Δ* is co-lethal with *cut3-477* and confers hypersensitivity to TBZ. Thus, *cut14-71* may affect the binding and/or the activity of condensin at the kinetochore in particular. *cut14-71* changes the glycine 397 of Cut14 into an aspartate. This residue is located at the very beginning of a coiled coil motif and is not conserved throughout evolution. Using the COILS program as a predictive tool (Lupas *et al.* 1991), we observed no disruption of the coiled-coil organization in the Cut14-71 mutant protein. Furthermore, Cut14 does not seem to directly contact the Pcs1/Mde4 monopolin complex (Tada *et al.* 2011). Thus, why *cut14-71* would have a specific impact on condensin at kinetochores remains enigmatic. The three other *slc* alleles of *cut14* (*slc85*, *slc90*, and *slc180*) all affect conserved residues located within the hinge domain. Notably, *slc90* affects a threonine residue conserved from bacteria to human and located near glycine patches clustered at the dimerization interface and essential for dimer formation and DNA binding (Hirano and Hirano 2002). Thus *slc85*, *slc90*, and *slc180* may weaken the interaction between the hinge domains of Cut3/Smc4 and Cut14/Smc2.

Arp9 is a subunit of the Swi/Snf and RSC (*i.e.*, remodels the structure of chromatin) chromatin remodeling complexes, whereas Snf21 is the ATP-dependent DNA helicase of RSC (Monahan *et al.* 2008). The fact that both *snf21-129* and *arp9-127* exhibit similar phenotypes, such as hypersensitivity to TBZ or HU, thermosensitivity, and the greatest frequency of chromatin bridges in anaphase at 32° among *slc* mutants, is consistent with their coexistence into functionally similar complexes. Arp9 may contribute to the association of RSC with chromatin as a heterodimer with Arp42 (Monahan *et al.* 2008), and lack of Arp42 or of the Snf5 subunit of Swi/Snf is synthetically sick in combination with *cut3-477* (Ryan *et al.* 2012). These data suggest that chromatin remodeling may play a role in chromosome condensation. ISWI chromatin remodeling complexes have been identified as major components of mitotic chromosomes assembled in *Xenopus laevis* egg extracts (MacCallum *et al.* 2002). However, the role of ISWI complexes in the formation of mitotic chromosome in this system appears rather limited. Furthermore, RSC has been implicated in multiple processes such as kinetochore function and recruitment of cohesin along chromosome arms (Hsu *et al.* 2003; Huang *et al.* 2004). Given the role of remodeling complexes as transcriptional coactivators, we cannot rule out the possibility that *snf21-129* and/or *arp9-127* mutations affect chromosome condensation indirectly. The hypersensitivity to HU of *snf2-129* and *arp9-127* mutants may reflect a defect in DNA replication during S phase, and the chromatin bridges exhibited by these mutants may stem, at least in part, from entry into mitosis with only partially replicated chromosomes. Alternatively, ATP-dependent chromatin remodeling may play an active role in the binding of condensin to “open” chromatin at promoters of active genes (see herein). Further work is required to determine whether or not the link we describe here between chromatin remodeling complexes and condensin is direct.

Cph2 is a zinc finger PHD domain protein associated with the Clr6 histone deacetylase within a complex called Clr6 II, which deacetylates histones mostly within coding regions of the genome (Nicolas *et al.* 2007). Clr6 II is the functional counterpart of budding yeast RPD3S, and RPD3-dependent deacetylation of histone H4-K5/K12 has been implicated in the recruitment of condensin onto rDNA in budding yeast in response to starvation (Tsang *et al.* 2007). Clr6 contributes to the regulation of condensin binding to retrotransposons in fission yeast through the deacetylation of H3-K56 (Tanaka *et al.* 2012), and the lack of Alp13, a nonessential subunit of Clr6 II, has been shown to cause chromosome segregation anomalies similar to those associated with defects in condensation (Nakayama *et al.* 2003). We found that, like *cph2Δ*, *alp13Δ* was colethal with *cut3-477* at 32° (see Figure S5). Thus, Cph2 may influence condensin association with chromatin as part of the Clr6 II histone deacetylase complex. Clr6 II may contribute to chromosome condensation by modulating the level of acetylation of nucleosomes, but possibly also of condensin itself, as suggested for the related cohesin complex (Kagami *et al.* 2011).

Ulp2 is a sumo-specific protease that plays a dual role in the cycle of sumoylation/desumoylation of proteins. Sumo (small ubiquitin-related modifier) is a reversible posttranslational protein modifier that is synthesized into an inactive proform. Ulp2 cleaves Sumo propeptide into a mature form that can be conjugated to target proteins, and, through its protease activity, also allows the de-sumoylation of target proteins (Geiss-Friedlander and Melchior 2007). Budding yeast *ULP2* has been identified as a multicopy suppressor of the *smc2-6* allele of condensin, and lack of *ULP2* is colethal with *smc4-1* (Bachellier-Bassi *et al.* 2008; Strunnikov *et al.* 2001). Thus, Ulp2 seems to play a positive role in chromosome condensation that is conserved in budding and fission yeasts. The targets of Ulp2 in this pathway may be multiple, including topoisomerases and/or condensin. Sumoylation of condensin subunits has been observed and is necessary for condensin recruitment to the rDNA repeats during anaphase (D’Amours *et al.* 2004; Takahashi *et al.* 2008). However, the underlying mechanism remains elusive. Ulp2 may play a positive role in this pathway because it seems required for the rDNA enrichment of Smc4-GFP (Strunnikov *et al.* 2001).

Nut2 is a component of the mediator complex, which physically associates with DNA binding transcription factors and RNA polymerase II. Through these contacts, mediator assists the recruitment of RNA polymerase II to genes promoters and integrates regulatory signals for transcription (Conaway and Conaway 2011). Mediator can stimulate or repress transcription and its subunits exhibit gene-specific effects. The origin of the chromosome segregation defects in *nut2Δ* cells remains undetermined. However, the fact that the lack of Nut2 improves chromosome segregation in *cut3-477* cells strongly suggests that the lack of Nut2 liberates a condensation activity that is limiting in a *cut3-477* background, and, therefore, that Nut2 plays a negative role in chromosome condensation. The restored growth of the *cut3-477 nut2Δ* double mutant at 34° compared with *cut3-477* most likely stems from the improved chromosome segregation. Nut2 may be required for the transcription of a negative regulator of chromosome condensation. Alternatively, given that transcription and condensin binding are antagonistic (Clemente-Blanco *et al.* 2009), an overall reduction in transcription caused by the lack of Nut2 may facilitate condensin binding to chromatin.

A recent report indicates that increasing cohesin occupancy on chromosomes by eliminating the destabilizing activity of Wapl leads to a substantial increase in the compaction of chromosomes (Tedeschi *et al.* 2013). Mediator and cohesin physically interacts and cooccupy active genes in human cells (Kagey *et al.* 2010). Thus, lack of Nut2 may compensate for condensin deficiency by increasing cohesin occupancy. However, we think this is unlikely because the lack of fission yeast Wapl (*wpl1Δ*) does not restore growth of *cut3-477* condensin mutant (Figure S6). Rather, given the overlap that seems to exist between condensin and cohesin for their loading onto chromosomes (D’Ambrosio *et al.* 2008), mediator may play a direct role in chromosome condensation through the recruitment of condensin onto chromatin, and Nut2 may negatively regulate this pathway.

One characteristic of condensin I and II complexes that recently emerged from genome-wide mapping experiments in budding and fission yeasts, *C. elegans*, chicken DT40 cells, and mouse embryonic stem cells is that both complexes are enriched at promoters of active genes (D’Ambrosio *et al.* 2008; Dowen *et al.* 2013; Kim *et al.* 2013; Kranz *et al.* 2013). The bacterial condensin of *Bacillus subtilis* also binds in the vicinity of highly expressed genes (Gruber and Errington 2009). Therefore, transcription somehow influences the binding of condensin complexes to chromosomes. Remarkably, four of the five “non-condensin” genes identified in this screen—*arp9*, *snf21*, *cph2*, and *nut2*—are implicated in transcription in eukaryotes. Swi/snf and RSC chromatin remodelers alter nucleosome positioning at gene promoters, thereby exposing DNA to the transcriptional machinery. Clr6 II/RPD3S deacetylates histones within the coding region of genes thereby promoting chromatin reformation in the wake of RNA polymerase II and prohibiting antisense transcription (Nicolas *et al.* 2007; Venkatesh *et al.* 2012). Furthermore, if we consider all known genetic interaction partners with *cut3* referenced in the pombase database (Wood *et al.* 2012), 28% (11/39) are linked to transcription. Although we cannot exclude the possibility of a role in pathways related to topo II and/or RCA, it is tempting to speculate that this enrichment in transcription cofactors reflects a link between condensin binding to chromosomes and high transcription rate. Local chromatin features established with transcription may play a role in condensin association with chromosome in eukaryotes. Overall, this genetic screen reinforces the idea of a tight functional interplay between condensin association with chromatin and transcriptional processes.

## Supplementary Material

Supporting Information
