# Supplementary material for: A Genetic Screen for Functional Partners of Condensin in Fission Yeast
Source: G3 (Bethesda). 2013 Dec 20;4(2):373–81. doi: 10.1534/g3.113.009621 (PMC3931570; doi:10.1534/g3.113.009621)
Supplement: Supporting Information [file supp_4_2_373__index.html]

A Genetic Screen for Functional Partners of Condensin in Fission Yeast — Supporting Information 

# A Genetic Screen for Functional Partners of Condensin in Fission Yeast

## Supporting Information for Robellet *et al.*, 2014

**Files in this Data Supplement:**

- Supporting Information - Figures S1-S6, Files S1-S4, and Table S1 (PDF, 817 KB)
- Figure S1 - Characterization of the thermosensitive *cut3-477* condensin mutant. (PDF, 545 KB)
- Figure S2 - *slc* mutations do not significantly reduce condensin and top2 steady state mRNA levels. (PDF, 421 KB)
- Figure S3 - Macroscopic phenotypes of *slc* mutants. (PDF, 541 KB)
- Figure S4 - Negative genetic interaction between *ulp2, cph2* or *arp9* and *cut3*. (PDF, 457 KB)
- Figure S5 - *cut3-477* confers hypersensitivity to the lack of *alp13*. (PDF, 430 KB)
- Figure S6 - Lack of Wpl1 does not suppress the thermosensitive growth phenotype of condensin mutants. (PDF, 332 KB)
- Table S1 - Strains used in this study (PDF, 327 KB)
- File S1 - Raw data sequencing *slc129* (.xlsx, 33 KB)
- File S2 - Raw data sequencing *slc174* (.xlsx, 31 KB)
- File S3 - Raw data sequencing *slc185* (.xlsx, 33 KB)
- File S4 - Raw data sequencing *sup122* (.xlsx, 31 KB)
