# Supplementary material for: A Genetic Screen for Functional Partners of Condensin in Fission Yeast
Source: G3 (Bethesda). 2013 Dec 20;4(2):373–81. doi: 10.1534/g3.113.009621 (PMC3931570; doi:10.1534/g3.113.009621)
Supplement: Supporting Information [file supp_g3.113.009621_009621SI.pdf]

G3·Genes | Genomes | Genetics

A genetic screen for functional partners of condensin in fission yeast

Xavier Robellet*[1,2], Lydia Fauque*[1], Pénélope Legros*, Esther Mollereau*, Stéphane Janczarski*, Morgane Thevenin*, Hugues Parrinello[§], Jean-Pierre Desvignes[§ 3] and Pascal Bernard*[4]

* LBMC, CNRS UMR 5239, Ecole Normale Supérieure de Lyon, Université Claude Bernard Lyon 1, Lyon, France, F-69364. § MGX-Montpellier GenomiX, Institut de Genomique Fonctionnelle, Montpellier, France, F-34094

1. Equal contribution.

2. Present address: IRIC, Université de Montréal. Montréal, CANADA, H3C 3J7.

3. Present address: U910 INSERM; Faculté de Médecine secteur Timone; Marseille, France

4. Corresponding author

DOI: 10.1534/g3.113.009621

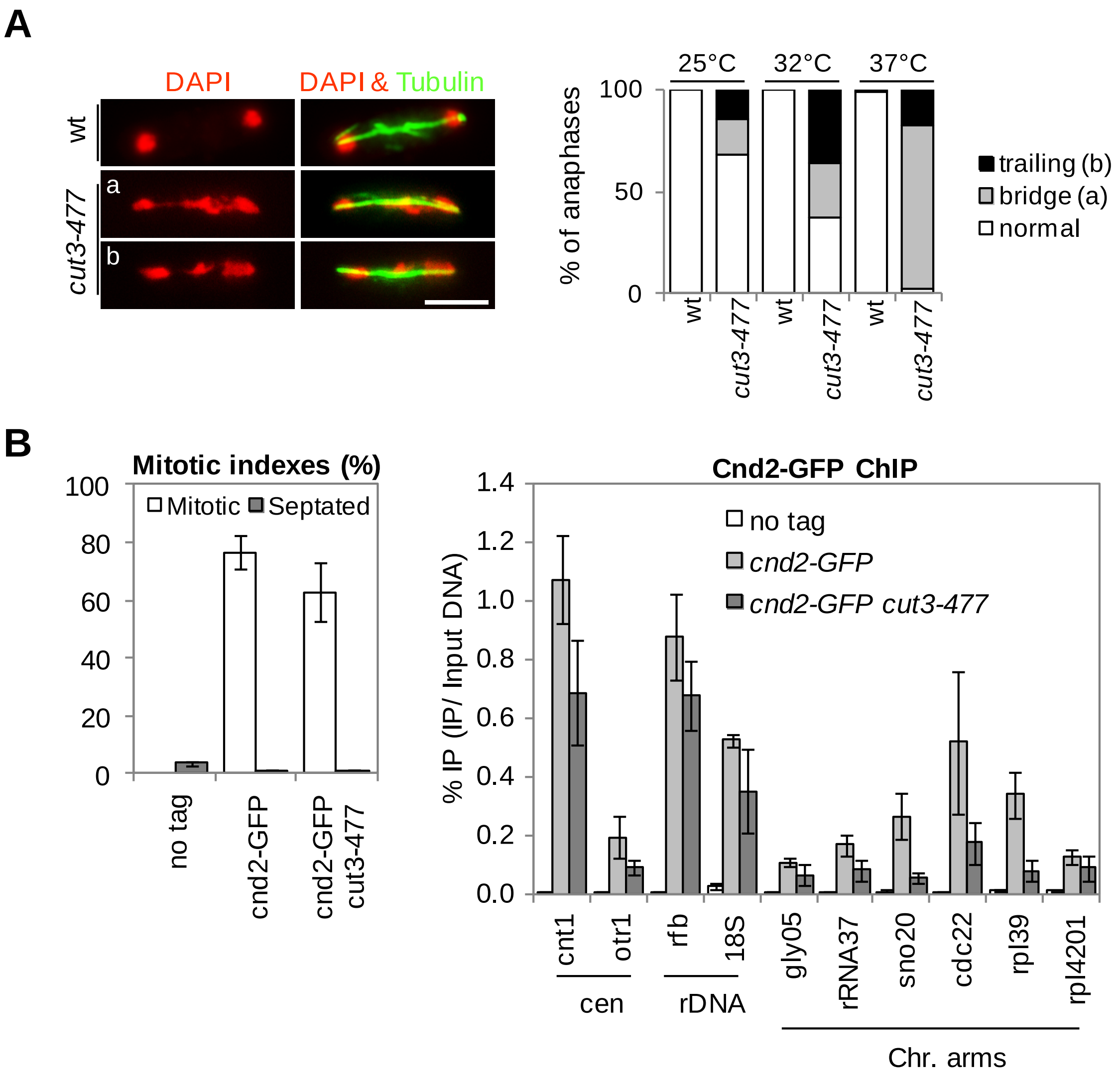

Figure S1 Characterization of the thermosensitive *cut3-477* condensin mutant

A. Cells grown at 25°C, 32°C or shifted from 25°C to 37°C and further incubated for 2 hours were fixed and processed for immunofluorescence against α-tubulin (Tubulin). DNA was stained with 4', 6'-diamidino-2-phenylindole (DAPI). Two representative *cut3-477* mutant cells exhibiting chromatin bridge (a) or trailing chromatin (b) are shown. Bar: 5 μm. Frequencies were determined by scoring at least 100 anaphase cells (mitotic spindle > 5 μm). **B.** Indicated strain were arrested in prometaphase at 17°C by using the *nda3-KM311* mutation and processed for ChIP against GFP. % IP correspond to the average and mean deviation obtained from 3 independent ChIPs. Mitotic arrests were assessed by scoring cells exhibiting nuclear Cnd2-GFP signals (Mitotic) and septated cells (Septated). Average and mean deviation calculated from three independent cultures are indicated.

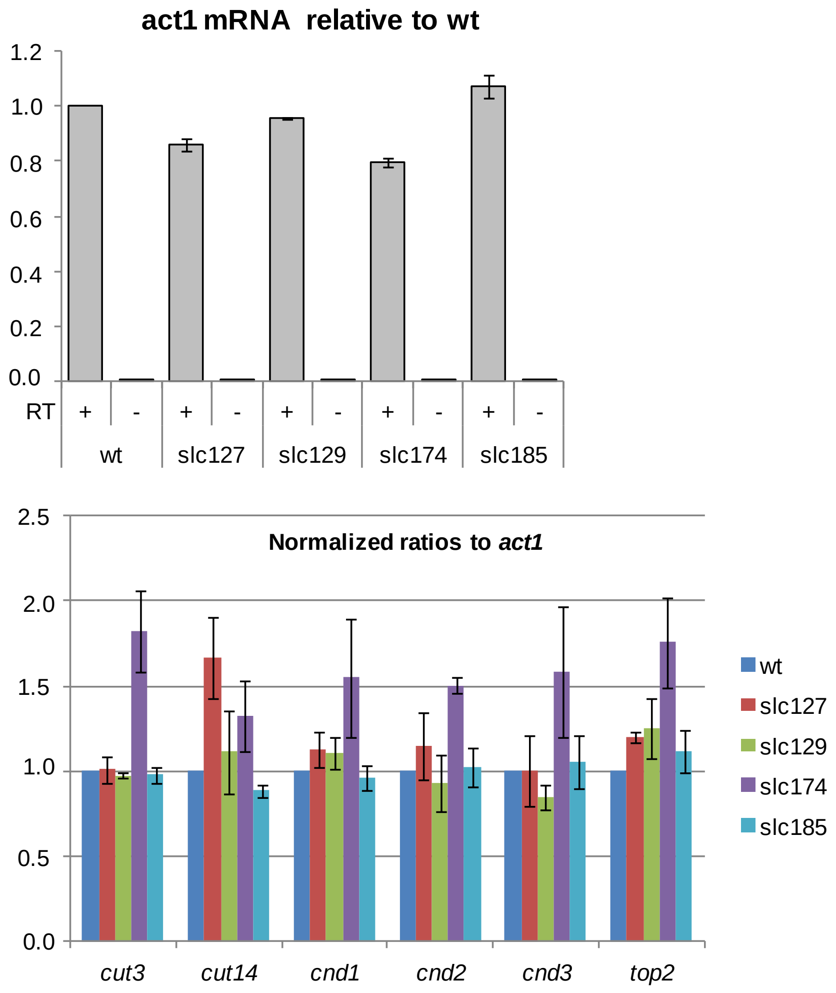

Figure S2 ***slc*** **mutations do not significantly reduce condensin and top2 steady state mRNA levels.**
Total RNA was extracted from cells exponentially growing at 32°C throughout the experiment. 500 ng of total RNA was reverse-transcribed in the presence (+) or absence (-) of Reverse Transcriptase (RT) and cDNAs were quantified by real time qPCR. Indicated values correspond to average and mean deviation from two independent experiments with two independent reverse transcriptions per experiment.

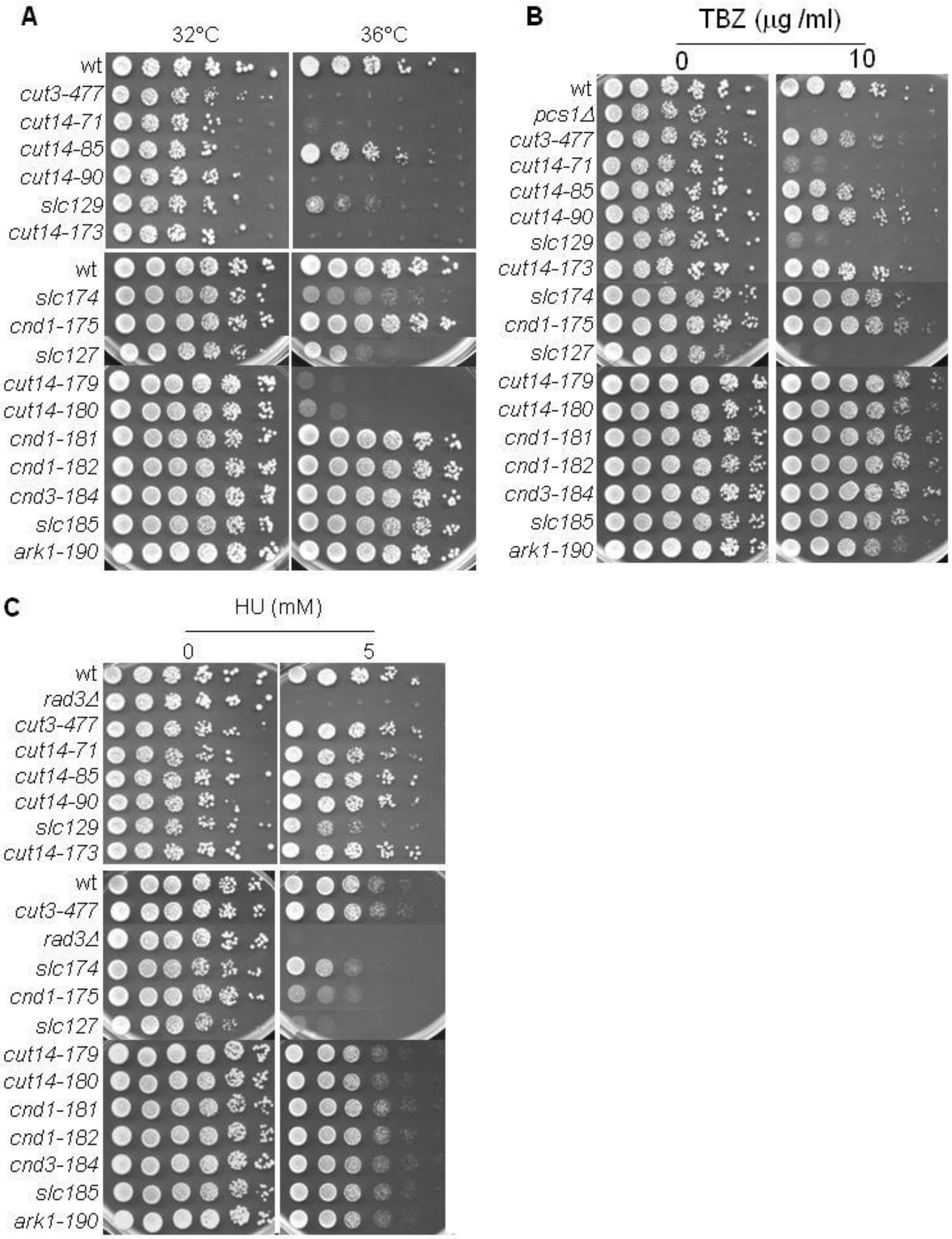

Figure S3 Macroscopic phenotypes of *slc* mutants

Strains of indicated genotypes were serially diluted and spotted onto indicated YES+A media. Growth in the presence of TBZ or HU was assessed at 32°C.

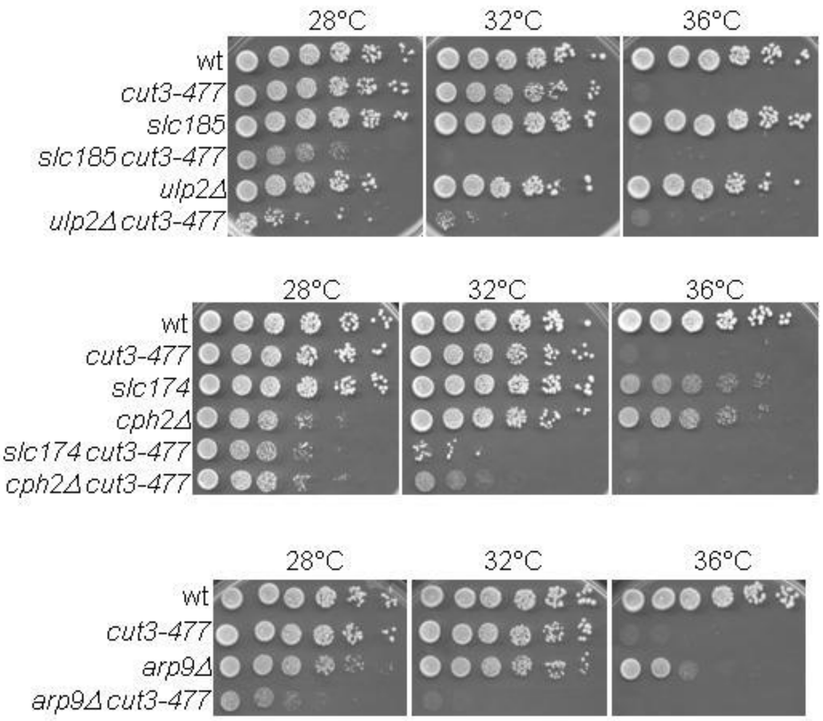

Figure S4 Negative genetic interaction between *ulp2*, *cph2* or *arp9* and *cut3*
Strains of indicated genotypes were serially diluted and spotted onto complete YES+A medium.

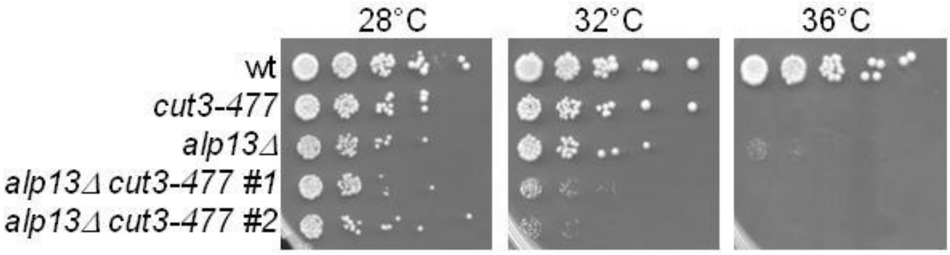

Figure S5 ***cut3-477*** **confers hypersensitivity to the lack of** ***alp13***
Strains of indicated genotypes were serially diluted and spotted on complete medium

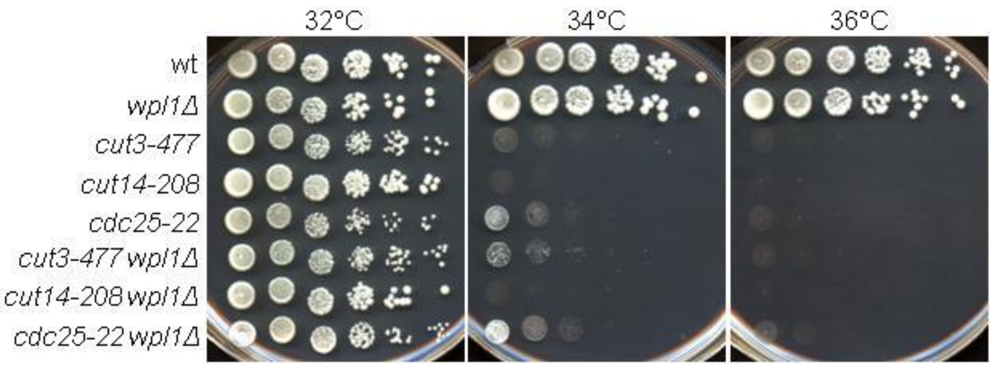

Figure S6 Lack of Wpl1 does not suppress the thermosensitive growth phenotype of condensin mutants
Strains of indicated genotypes were serially diluted and spotted onto complete medium. Plates were incubated for 4 days. Thermosensitive *cdc25-22* was used as a specificity control.

Files S1-S4

Available for download as Excel files at http://www.g3journal.org/lookup/suppl/doi:10.1534/g3.113.009621/-/DC1

File S1 Raw data sequencing *slc129*

File S2 Raw data sequencing *slc174*

File S3 Raw data sequencing *slc185*

File S4 Raw data sequencing *sup122*

Table S1 Strains used in this study

**number**	**genotype**	**origin**
**LY113**	*h- leu1-32 ura4D18 ade6-210*	Lab stock
**LY45**	*h- leu1-32 ura4D18 cut3-477*	YGRC
**LY270**	*h- leu1-32 ura4D18 nda3-KM311*	Lab stock
**LY1802**	*h- leu1-32 ura4D18 or DS/E nda3-KM311 cnd2-gfp-LEU2*	YGRC
**LY2046**	*h- leu1-32 ura4D18 or DS/E cut3-477 nda3-KM311 cnd2-gfp-LEU2*	Lab stock
**LY78**	*h- leu1-32 cut3-477 pREP41XL-cut3-LEU2*	Lab stock
**LY69**	*h- leu1-32 cut3-477 pREP41XL-LEU2*	Lab stock
**LY42**	*h- leu1-32 top2-250*	YGRC
**LY75**	*h- leu1-32 cut3-477 top2-250 pREP41XL-cut3-LEU2*	Lab stock
**LY1064**	*h- leu1-32 ura4D18 ade6-210 slc71*	Lab stock
**LY862**	*h- leu1-32 ura4D18 ade6-210 slc85*	Lab stock
**LY646**	*h- leu1-32 ura4D18 or DS/E slc90*	Lab stock
**LY1241**	*h+ leu1-32 ura4D18 or DS/E ade6-210 slc127*	Lab stock
**LY962**	*h- leu1-32 ura4D18 or DS/E ade6-216 slc129*	Lab stock
**LY858**	*h- leu1-32 ura4D18 or DS/E ade6-216 slc173*	Lab stock
**LY1259**	*h+ leu1-32 ade6-210 slc174*	Lab stock
**LY1261**	*h+ leu1-32 ura4D18 or DS/E ade6-210 slc175*	Lab stock
**LY1250**	*h- leu1-32 ura4D18 or DS/E ade6-210 slc179*	Lab stock
**LY1252**	*h- leu1-32 ura4D18 or DS/E ade6-210 slc180*	Lab stock
**LY1264**	*h- leu1-32 ura4D18 or DS/E ade6-210 slc181*	Lab stock
**LY1266**	*h- leu1-32 ura4D18 or DS/E ade6-210 slc182*	Lab stock
**LY1268**	*h- leu1-32 ura4D18 or DS/E ade6-210 slc184*	Lab stock
**LY1269**	*h- leu1-32 ura4D18 or DS/E ade6-210 slc185*	Lab stock
**LY1271**	*h- leu1-32 ade6-210 slc190*	Lab stock
**LY904**	*h- leu1-32 ura4D18 or DS/E ade6-216 cut3-477 sup122*	Lab stock
**LY480**	*h- ura4D18 rad3Δ::ura4+*	YGRC
**LY28**	*h- leu1-32 ura4D18 ade6-210 pcs1Δ::ura4+*	JP Javerzat
**LY2625**	*h+ leu1-32 ura4D18 ade6-210 arp9Δ::NatR*	Lab stock
**LY2601**	*h+ leu1-32 ura4D18 ade6-216 cph2Δ::NatR*	Lab stock
**LY2602**	*h+ leu1-32 ura4D18 ade6-216 ulp2Δ::NatR*	Lab stock
**LY2626**	*h+ leu1-32 ura4D18 ade6-216 nut2Δ::NatR*	Lab stock
**LY1948**	*h- leu1-32 ura4D18 ade6-210 alp13Δ::NatR*	Lab stock
**LY662**	*h+ leu1-32 ura4D18 wpl1Δ::kanR*	JP Javerzat
