# Supplementary material for: A Genetic Screen for Functional Partners of Condensin in Fission Yeast
Source: G3 (Bethesda). 2013 Dec 20;4(2):373–81. doi: 10.1534/g3.113.009621 (PMC3931570; doi:10.1534/g3.113.009621)
Supplement: Supporting Information [file supp_g3.113.009621_FigureS1.pdf]

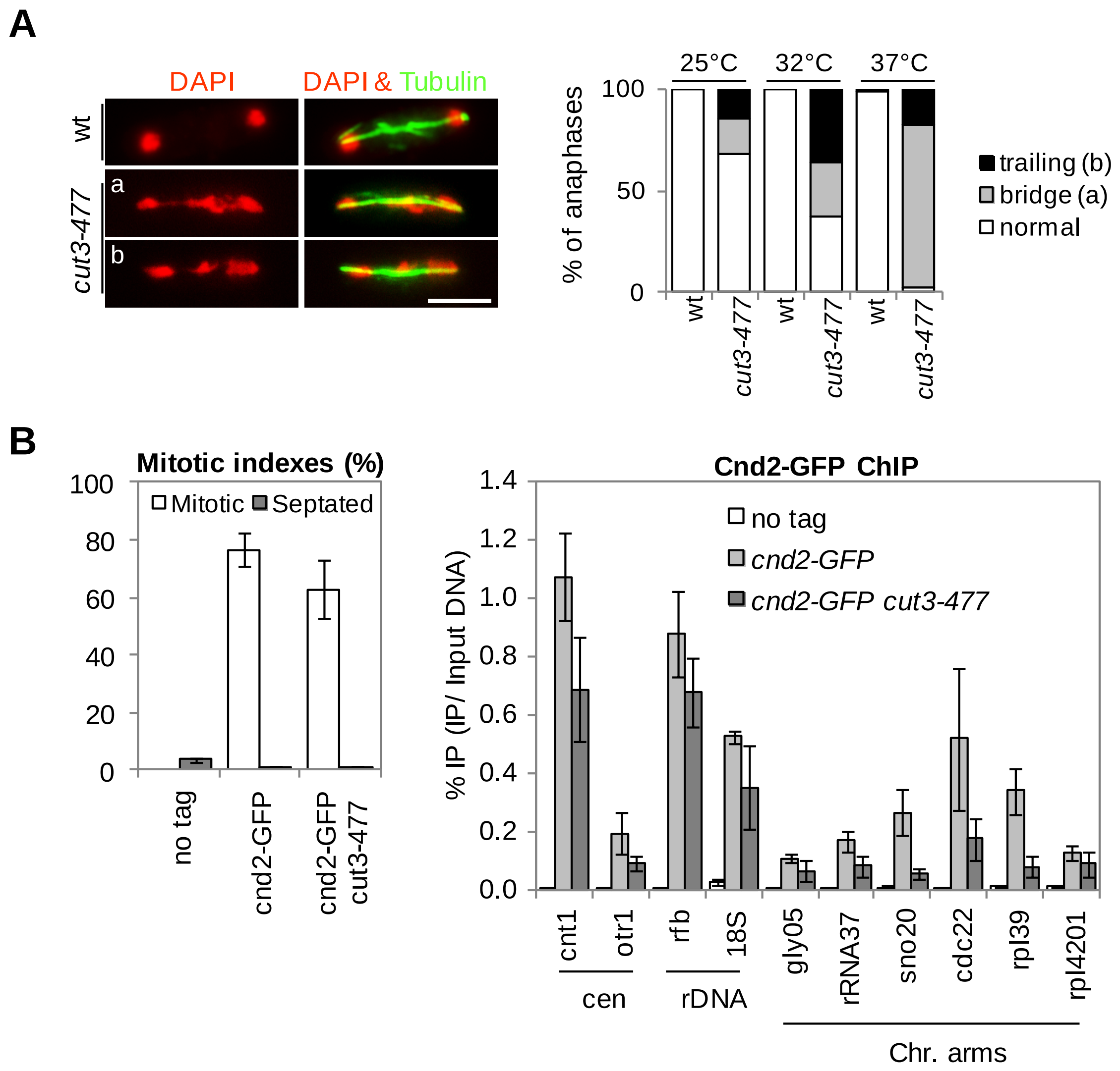

Figure S1 Characterization of the thermosensitive *cut3-477* condensin mutant

A. Cells grown at 25°C, 32°C or shifted from 25°C to 37°C and further incubated for 2 hours were fixed and processed for immunofluorescence against α-tubulin (Tubulin). DNA was stained with 4', 6'-diamidino-2-phenylindole (DAPI). Two representative *cut3-477* mutant cells exhibiting chromatin bridge (a) or trailing chromatin (b) are shown. Bar: 5 μm. Frequencies were determined by scoring at least 100 anaphase cells (mitotic spindle > 5 μm). **B.** Indicated strain were arrested in prometaphase at 17°C by using the *nda3-KM311* mutation and processed for ChIP against GFP. % IP correspond to the average and mean deviation obtained from 3 independent ChIPs. Mitotic arrests were assessed by scoring cells exhibiting nuclear Cnd2-GFP signals (Mitotic) and septated cells (Septated). Average and mean deviation calculated from three independent cultures are indicated.
