# Supplementary material for: A Genetic Screen for Functional Partners of Condensin in Fission Yeast
Source: G3 (Bethesda). 2013 Dec 20;4(2):373–81. doi: 10.1534/g3.113.009621 (PMC3931570; doi:10.1534/g3.113.009621)
Supplement: Supporting Information [file supp_g3.113.009621_FigureS2.pdf]

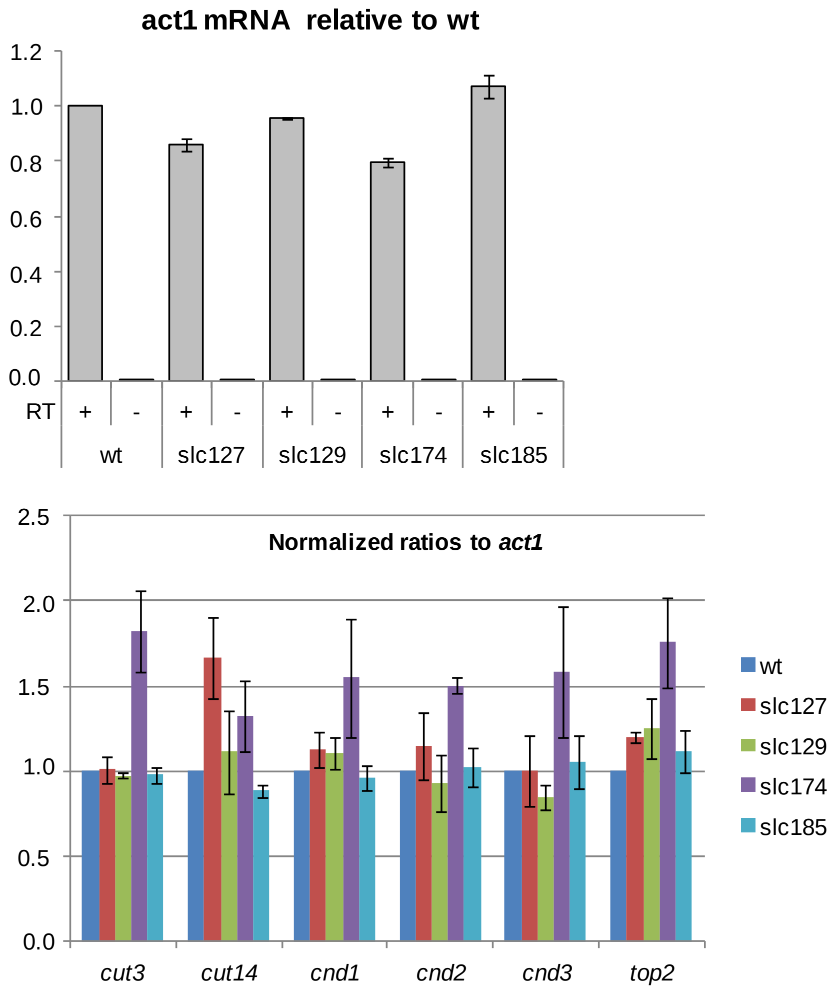

Figure S2 ***slc*** **mutations do not significantly reduce condensin and top2 steady state mRNA levels.**
Total RNA was extracted from cells exponentially growing at 32°C throughout the experiment. 500 ng of total RNA was reverse-transcribed in the presence (+) or absence (-) of Reverse Transcriptase (RT) and cDNAs were quantified by real time qPCR. Indicated values correspond to average and mean deviation from two independent experiments with two independent reverse transcriptions per experiment.
