# Supplementary material for: A Genetic Screen for Functional Partners of Condensin in Fission Yeast
Source: G3 (Bethesda). 2013 Dec 20;4(2):373–81. doi: 10.1534/g3.113.009621 (PMC3931570; doi:10.1534/g3.113.009621)
Supplement: Supporting Information [file supp_g3.113.009621_FigureS3.pdf]

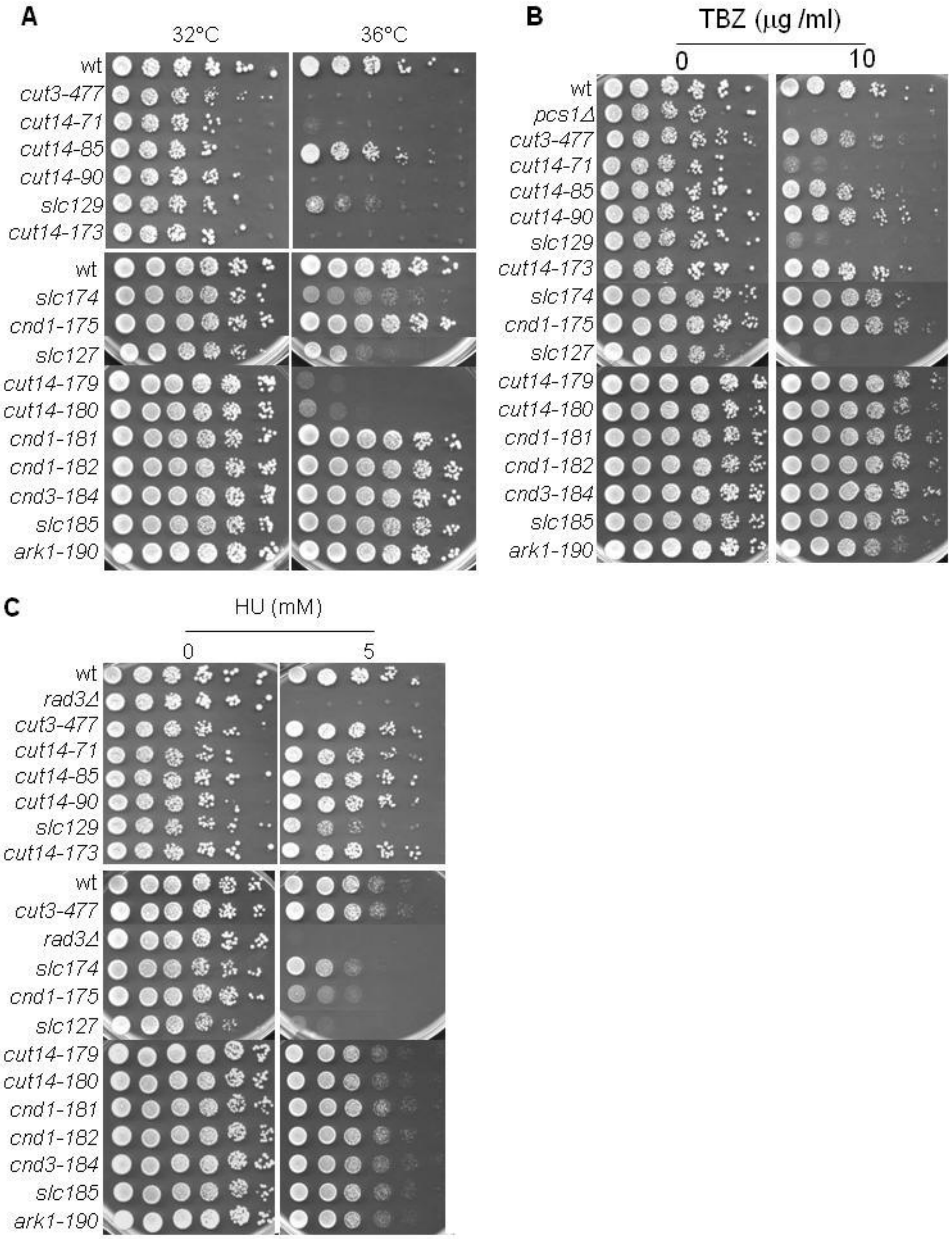

Figure S3 Macroscopic phenotypes of *slc* mutants

Strains of indicated genotypes were serially diluted and spotted onto indicated YES+A media. Growth in the presence of TBZ or HU was assessed at 32°C.
