# Supplementary material for: A Genetic Screen for Functional Partners of Condensin in Fission Yeast
Source: G3 (Bethesda). 2013 Dec 20;4(2):373–81. doi: 10.1534/g3.113.009621 (PMC3931570; doi:10.1534/g3.113.009621)
Supplement: Supporting Information [file supp_g3.113.009621_FigureS5.pdf]

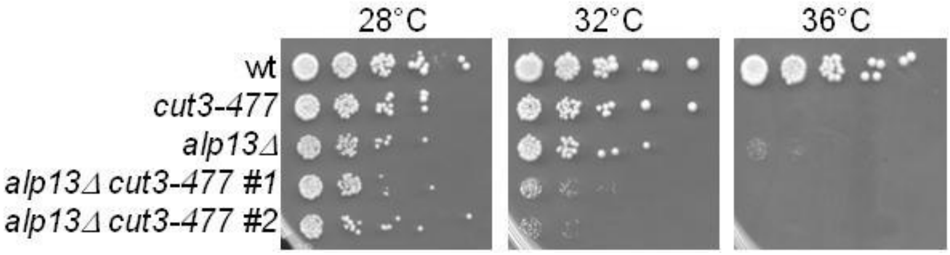

Figure S5 ***cut3-477* confers hypersensitivity to the lack of *alp13***
Strains of indicated genotypes were serially diluted and spotted on complete medium
