# Supplementary material for: A Genetic Screen for Functional Partners of Condensin in Fission Yeast
Source: G3 (Bethesda). 2013 Dec 20;4(2):373–81. doi: 10.1534/g3.113.009621 (PMC3931570; doi:10.1534/g3.113.009621)
Supplement: Supporting Information [file supp_g3.113.009621_FigureS6.pdf]

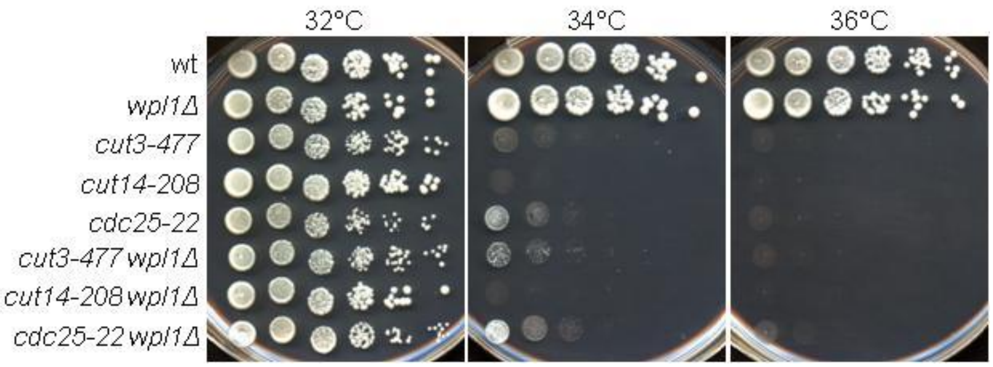

Figure S6   Lack of Wpl1 does not suppress the thermosensitive growth phenotype of condensin mutants
Strains of indicated genotypes were serially diluted and spotted onto complete medium. Plates were incubated for 4 days. Thermosensitive *cdc25-22* was used as a specificity control.
