# Supplementary material for: A Genetic Screen for Functional Partners of Condensin in Fission Yeast
Source: G3 (Bethesda). 2013 Dec 20;4(2):373–81. doi: 10.1534/g3.113.009621 (PMC3931570; doi:10.1534/g3.113.009621)
Supplement: Supporting Information [file supp_g3.113.009621_TableS1.pdf]

Table S1 Strains used in this study

number	genotype	origin
**LY113**	*h- leu1-32 ura4D18 ade6-210*	Lab stock
**LY45**	*h- leu1-32 ura4D18 cut3-477*	YGRC
**LY270**	*h- leu1-32 ura4D18 nda3-KM311*	Lab stock
**LY1802**	*h- leu1-32 ura4D18 or DS/E nda3-KM311 cnd2-gfp-LEU2*	YGRC
**LY2046**	*h- leu1-32 ura4D18 or DS/E cut3-477 nda3-KM311 cnd2-gfp-LEU2*	Lab stock
**LY78**	*h- leu1-32 cut3-477 pREP41XL-cut3-LEU2*	Lab stock
**LY69**	*h- leu1-32 cut3-477 pREP41XL-LEU2*	Lab stock
**LY42**	*h- leu1-32 top2-250*	YGRC
**LY75**	*h- leu1-32 cut3-477 top2-250 pREP41XL-cut3-LEU2*	Lab stock
**LY1064**	*h- leu1-32 ura4D18 ade6-210 slc71*	Lab stock
**LY862**	*h- leu1-32 ura4D18 ade6-210 slc85*	Lab stock
**LY646**	*h- leu1-32 ura4D18 or DS/E slc90*	Lab stock
**LY1241**	*h+ leu1-32 ura4D18 or DS/E ade6-210 slc127*	Lab stock
**LY962**	*h- leu1-32 ura4D18 or DS/E ade6-216 slc129*	Lab stock
**LY858**	*h- leu1-32 ura4D18 or DS/E ade6-216 slc173*	Lab stock
**LY1259**	*h+ leu1-32 ade6-210 slc174*	Lab stock
**LY1261**	*h+ leu1-32 ura4D18 or DS/E ade6-210 slc175*	Lab stock
**LY1250**	*h- leu1-32 ura4D18 or DS/E ade6-210 slc179*	Lab stock
**LY1252**	*h- leu1-32 ura4D18 or DS/E ade6-210 slc180*	Lab stock
**LY1264**	*h- leu1-32 ura4D18 or DS/E ade6-210 slc181*	Lab stock
**LY1266**	*h- leu1-32 ura4D18 or DS/E ade6-210 slc182*	Lab stock
**LY1268**	*h- leu1-32 ura4D18 or DS/E ade6-210 slc184*	Lab stock
**LY1269**	*h- leu1-32 ura4D18 or DS/E ade6-210 slc185*	Lab stock
**LY1271**	*h- leu1-32 ade6-210 slc190*	Lab stock
**LY904**	*h- leu1-32 ura4D18 or DS/E ade6-216 cut3-477 sup122*	Lab stock
**LY480**	*h- ura4D18 rad3Δ::ura4+*	YGRC
**LY28**	*h- leu1-32 ura4D18 ade6-210 pcs1Δ::ura4+*	JP Javerzat
**LY2625**	*h+ leu1-32 ura4D18 ade6-210 arp9Δ::NatR*	Lab stock
**LY2601**	*h+ leu1-32 ura4D18 ade6-216 cph2Δ::NatR*	Lab stock
**LY2602**	*h+ leu1-32 ura4D18 ade6-216 ulp2Δ::NatR*	Lab stock
**LY2626**	*h+ leu1-32 ura4D18 ade6-216 nut2Δ::NatR*	Lab stock
**LY1948**	*h- leu1-32 ura4D18 ade6-210 alp13Δ::NatR*	Lab stock
**LY662**	*h+ leu1-32 ura4D18 wpl1Δ::kanR*	JP Javerzat
